# The Experiences of Social Connection and Isolation in Adults With Acquired Brain Injury: A Qualitative Systematic Review and Metasynthesis

**DOI:** 10.1111/hex.70420

**Published:** 2025-09-08

**Authors:** Jordan Ayden, María‐José Bracho‐Ponce, Julia Ajayi, Sarah Hanson, Fergus Gracey

**Affiliations:** ^1^ Department of Clinical Psychology and Psychological Therapies, Norwich Medical School University of East Anglia Norwich UK; ^2^ Centro de Estudios en Neurociencia Humana y Neuropsicología Universidad Diego Portales Santiago Chile; ^3^ School of Health Sciences University of East Anglia Norwich UK

**Keywords:** acquired brain injury, qualitative, social connection, social isolation

## Abstract

**Background:**

During recovery from an acquired brain injury (ABI), social isolation is a common experience that can lead to adverse outcomes. Although social connection is known to play a critical role in alleviating these effects, the ways in which ABI survivors experience and prioritise connection and isolation are not well understood. This review aims to understand how these concepts are perceived, identify the valued outcomes, and examine the social contexts that shape these experiences.

**Method:**

Peer‐reviewed qualitative articles published before January 2025 were identified from a search of six databases and additional complementary searches. These encompassed the terms ‘acquired brain injury’, ‘social connection and/or isolation’ and ‘qualitative’. Of 4651 papers identified, 37 were included. Each paper was categorised for the analytic process according to its relevance, resonance and rigour, with rigour assessed using the JBI standardised critical appraisal instrument. Thomas and Harden's ‘thematic synthesis’ framework was used to generate a metasynthesis rooted in a critical realist philosophy.

**Results:**

Social isolation is a widespread issue characterised by loss, which originates from ABI‐related impairments that hinder social engagement. Communication difficulties, stigma and other structural barriers exacerbate feelings of disconnection. Key elements of social connection included understanding, acceptance, emotional support, reciprocity and shared experiences. Relationships that embodied these qualities provided a sense of belonging, security and purpose, which encouraged individuals to redefine their identity and values, alleviating feelings of isolation.

**Conclusion:**

This review demonstrates that social connection and isolation post‐ABI stem from a variety of complex interpersonal and environmental factors. Future research should prioritise measuring and enhancing meaningful connections by focusing on relational quality, accessibility, and reducing systemic barriers, including stigma.

**Patient or Public Contribution:**

The project's focus was developed in consultation with members of the public who have lived experience with an ABI. A PPI member also supported the entire review process.

## 1 Introduction

An acquired brain injury (ABI) refers to a non‐degenerative injury to the brain that takes place after birth. While traumatic brain injuries (TBIs) are caused by external events like falls or accidents, non‐TBIs result from internal processes such as infections, strokes or tumours [1]. ABIs are a primary cause of disability and death [2], with approximately 69 million people sustaining one each year globally [3]. In the United Kingdom, it is estimated that over 1.3 million people live with an ABI, and hospital admissions occur every 90 s due to such injuries. Over the past 18 years, the incidence of ABIs has risen by 12% [4], a trend expected to continue due to the ageing population [5]. The economic impact of ABIs on the United Kingdom is significant, with annual costs reaching £43 billion [6]. Given the rising prevalence of ABIs and the associated financial burden, it is crucial that treatment and rehabilitation efforts are effective.

Rehabilitation prioritises reducing impairment and enhancing functional independence [7]. However, individuals with an ABI have diverse needs beyond this scope [8, 9]. Social connection and isolation are consistently identified as central concerns [10] that impact quality of life post‐ABI [7]. Nevertheless, these relational needs are often overlooked in the conventional western medical rehabilitation approach. Effective treatment and rehabilitation require prioritising outcomes that hold significance for those with an ABI. By incorporating the perspectives of people with an ABI into selecting valued outcomes, rehabilitation can be better designed to meet their holistic needs [11].

Researchers have distinguished between perceived isolation, an individual's subjective experience of feeling isolated, and objective isolation, defined by limited social interactions and network size [12]. Meanwhile, social connection pertains to the feeling of belonging and the personal and meaningful connections individuals experience in social interactions and relationships [13]. Social connection and isolation are distinct but interrelated constructs, both shown to significantly influence physical and mental health [14].

Both perceived [15] and objective isolation are heightened [16], and social connection is reduced post‐ABI [17, 18]. This is attributed to ABI‐related deficits, which make social interactions and maintaining and developing relationships more difficult [19, 20]. For example, functional, physical and cognitive impairments may make it more challenging for individuals to travel for community, social or vocational activities. Fatigue, cognitive deficits and negative reactions from others further complicate social interactions, even virtually or at home [21, 22]. More than 60% of ABI survivors report having no interactions outside of healthcare staff and family members, emphasising the profound impact of an ABI on social participation [23]. This isolation reduces quality of life [7] and harms mental and physical health [24], particularly by contributing to depression [20]. These impede functional progress [25] and are linked to poorer rehabilitation outcomes [26] and a lower likelihood of returning to work [27], all of which contribute to the substantial economic cost of ABIs [6]. Conversely, social connectedness is associated with reduced depressive symptoms [28], increased resilience, a greater sense of purpose, and more significantly, rapid functional improvements after injury [29, 30, 31]. Therefore, promoting social connection could mitigate the adverse effects of isolation and reduce the wider economic burden of prolonged rehabilitation, such as increased healthcare costs and loss of productivity.

Promoting social connection requires the ability to meaningfully assess it. However, most existing outcome measures are not validated for use in ABI populations, and the measures do not adequately capture individuals' nuanced and diverse social experiences post‐ABI. Social connection is often inferred through proxy indicators, including social network size, which may not reflect the context, manner and value of social connection [32], nor sensitively capture social change. This presents a challenge in identifying individuals' social needs and evaluating the effectiveness of interventions [33]. Tailored and validated measures of social connection and isolation are therefore required [34] to assess outcomes that are relevant [35] and meaningful to individuals with ABI [11]. Gaining insight into how ABI survivors experience and value social connection and isolation is essential to deepen our understanding of these concepts and identify the most relevant and meaningful outcomes, conceptualised here as priority areas for ABI survivors.

The present review synthesised qualitative research that explored the experiences of adults with an ABI regarding social connection and isolation following their injury. It aims to articulate what these concepts mean to individuals, identify the social outcomes that are most meaningful, and examine the social contexts that shape these experiences. By providing a rich, descriptive understanding of social connection and isolation, this review aims to inform future intervention development tailored to the lived experience of adults with ABI. The research questions were:

1. How do adults with ABI perceive and experience social connection and isolation following their injury?
2. What specific outcomes related to social connection and isolation are important to adults with an ABI?
3. What are the particular social contexts that influence the experience of social connection and isolation for individuals with an ABI?

## 2 Methods

The review protocol was registered on the International Prospective Register of Systematic Reviews (PROSPERO; CRD42024585118) in October 2024, and the Preferred Reporting Items for Systematic Reviews and Meta‐Analyses (PRISMA) guidelines were followed.

### 2.1 Inclusion Criteria

The inclusion criteria targeted research exploring social connection and isolation in adults aged 18+ with an ABI, and studies involving mixed populations were included only when findings specific to the ABI group were clearly identifiable. Included were peer‐reviewed, primary qualitative papers or qualitative components of mixed‐methods studies, conducted in post‐acute settings in any location.

### 2.2 Exclusion Criteria

Papers that included participants with mild TBIs or concussions were excluded due to their differing effects and recovery trajectories [36].

### 2.3 Search Strategy

The search strategy was designed in collaboration with a specialist health librarian to identify papers that referred to the three key elements of the review: ‘acquired brain injury’, ‘social isolation and/or connection’ and ‘qualitative’. This was adapted for each database searched: CINAHL (EBSCOhost interface), PsycINFO (EBSCOhost interface), Medline (OVID interface), Embase (OVID interface), the Allied and Complementary Medicine Database (AMED; EBSCOhost interface) and the Web of Science Core Collection (Clarivate interface). The full search strategy for each database is provided in Supporting File 1. Reference lists of included studies and relevant reviews were screened to identify additional eligible studies. Searches were limited to English‐language publications up to January 2025.

### 2.4 Search Outcome and Selection Process

**Figure 1:**
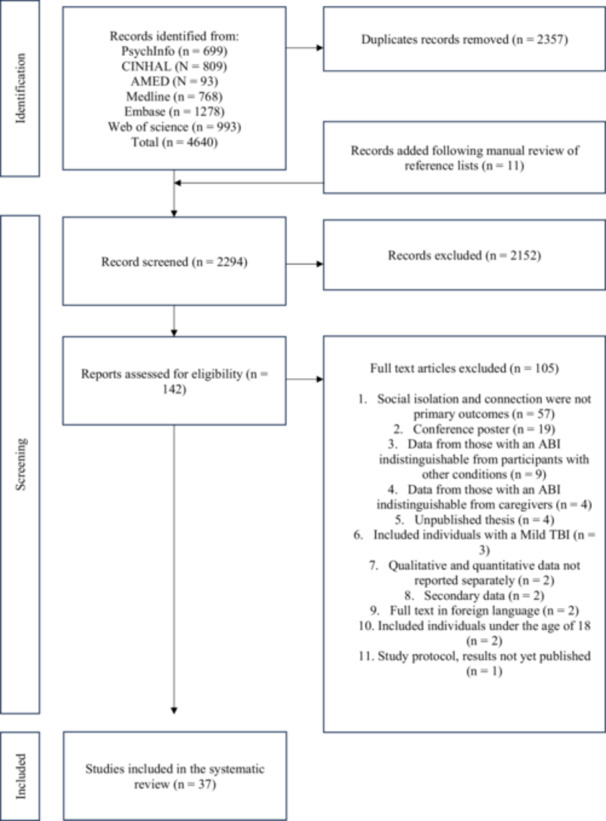
PRISMA flow diagram.

The database search identified 4640 papers, with 11 found through complementary searches. After removing duplicates, 2294 studies were screened through the Rayyan platform. The PRISMA flow diagram [37] (Figure 1) illustrates the number of studies excluded at each stage, with justification. A pilot test was conducted, where J.A. and M.B. independently screened the titles and abstracts of 40 papers to establish a clear understanding of the criteria for full‐text review. After reaching an agreement through one discussion, J.A. screened all remaining titles and abstracts, with M.B. reviewing 10% to ensure consistency. Full agreement was achieved. Subsequently, J.A. assessed all full texts against the inclusion and exclusion criteria, while M.B. independently reviewed a random 10% (*n* = 14) for reliability, with no disagreements arising. Due to practical constraints, double screening of all full texts was not feasible.

### 2.5 Data Extraction

Data was extracted by J.A., and half was randomly checked for accuracy by S.H. and F.G. No discrepancies were identified, requiring no changes. For analysis, only direct quotes from participants in the finding sections pertaining to social connection and isolation were used to minimise author interpretation and keep the voices of those with lived experience central to the synthesis. Key study characteristics were extracted into Excel (Table 1).

**Table 1:** Data extraction.

Author, year, country	Participants (including socio‐economic and ethnicity data where available)	Research aim	Study methods	Method adaptations for inclusion	Key findings in relation to social connection and isolation	Authors' suggestions	JBI rating	Quality of contribution to the review
[38], Ireland	14 participants (8 men and 6 women) with ABI. The age range was 35–64, and the time since injury varied between 7.22 and 8.72 years. 6 participants were employed, 1 was a student and 7 were unemployed. Ethnicity information not reported.	Explore the experience of living with an ABI in individuals who report higher or lower PTG.	Semi‐structured interviews analysed using reflexive thematic analysis. A critical realist, multi‐method design was used, splitting groups by a quantitative PTG measure. This forms part of a longitudinal study with the same cohort of people with ABI followed up after 7 years.	In the case of a participant who presented with speech difficulties, the interview was completed using a combination of video‐teleconferencing and email. Participants were encouraged from the outset of each interview, to ask for breaks as necessary.	Four themes were identified, one of which was linked to social connection: Growing in the social world: ‘you need to have that social connection’.	Longitudinal research examining the obstacles facing individuals with ABI for adjustment and PTG could help improve clinical services. It is also important to understand the role of the social environment in developing PTG: Could PTG, acceptance, and self‐continuity or connection be outcomes of meaningful social connection?	9	Central
[71], the United States	27 participants (9 men and 18 women) with post‐stroke aphasia. The mean age was 58.8 years, and the time since onset ranged between 6 and 306 months. No ethnicity or socio‐economic data reported.	To better understand the perspectives of people with aphasia on why some friendship bonds remain strong and some do not and to explore how age and aphasia severity shape views on friendship.	Semi‐structured interviews analysed using framework analysis and reflexive thematic analysis. Philosophical approach not stated.	Implemented supported conversation strategies, such as allowing ample time for the interviewee to respond, providing interviewees with written versions of questions or key words, repeating questions or speaking turns, seeking confirmation of previous responses, and encouraging multimodal responses (e.g., gesturing, writing, using the chat function, using text‐to‐speech applications and retrieving photos from their smartphones).	Four major themes concerning how friend relationships had been impacted by aphasia were identified: Not all bonds have the same chance of surviving the onset of aphasia (long‐standing friendships are more likely to remain active; work‐based relationships are difficult to maintain; connections with people who have experienced health crises tend to remain strong); people with aphasia's closest friends took active steps to keep relationships strong (reaching out and staying in touch helped a lot; adapting activities to increase accessibility kept friendships alive; providing highly practical forms of support leads to stronger bonds); if friends knew some basic information about aphasia, bonds would stay stronger (an elementary knowledge of aphasia would help friends stick around; relationships with friends who used supportive communication strategies remained satisfying and active; technology plays a large role in helping stay connected to friends); positive affective aspects of friendship play an important role in keeping bonds strong (maintaining reciprocity is important; friends who valued and celebrated recovery were highly appreciated; empathy was a key ingredient in healthy friendships).	Future research should explore how age and severity interact to influence social connectedness or isolation. Future studies should aim for a more diverse and inclusive participant pool in race, ethnicity, sexual orientation and other identity factors, which are known to affect friendships.	7	Core
[39], the United Kingdom	24 participants (15 men and 9 women) with dysarthria as a result of stroke. The mean age was 63.5 years, and the time since stroke varied between 2 and 34 months. 1 participant lived in very high deprivation and 7 in very low deprivation. No further socio‐economic or ethnicity data reported. The same sample as [40] was used.	Explore the impact of dysarthria on social participation following stroke.	Semi‐structured interviews analysed thematically adopting a constant comparative methodology. Philosophical orientation not stated. Caregiver data was also collected but reported separately.	None reported.	Two themes were identified: Social isolation (I won't talk to someone for days; Nobody could understand what I was saying; They'll speak to her first; Be normal, please; You feel as if you're left out; I hate the phone; I go to the self‐service now; Say ‘Aye’ or ‘No’, that's all; I was ordered to go to that); Identity (‘It wasn't my voice’; ‘Bill that's had a stroke’; Stigmatising markers).	Future work should consider the psychosocial impacts experienced by those with severe dysarthria. Additionally, research must design, develop and evaluate interventions that aim to address the long‐term issues raised for both the individual and their carers.	3	Central
[41], Australia	25 participants (13 men and 12 women) with moderate‐to‐severe post‐stroke aphasia. The mean age was 63.2, and the time since onset ranged between 24 and 299 months. 16 participants had a combined annual income of <$30,000 AUD and 9 of ≥ 30,000 AUD. No ethnicity information.	Explore the perspectives of individuals with aphasia on the role of friendship in living successfully with aphasia.	Two qualitative interviews with each participant analysed using thematic analysis. Following the first interview each participant was provided with a disposable camera and was asked to take photographs representing what living successfully with aphasia meant to them. These were discussed during the second interview. A phenomenological approach was adopted.	Interviews adhered to the guidelines for Supportive Conversation for Adults with Aphasia and used aphasia‐friendly conversational support materials based on principles. Photos were also used to aid conversations.	The interviews revealed interviews revealed three over‐arching themes: Living with changes in friendships (Loss of friendship; Friends' lack of understanding about aphasia; Increased dependency and lost opportunities for contact with friends; Difficulties talking with friends; Desire for more friends and increased social contact; Increased appreciation for friends; New friendships and being proactive), good times together and support from friends (Spending time with friends and doing things together; Laughter and engaging in positive interactions with friends; Emotional support from friends), and the importance of stroke and aphasia friends (Mutual understanding and ease of communication; Helping each other).	Little attention has been given to education about aphasia and conversation partner training for friends or other interventions that address friendship in the context of aphasia. Findings from this study highlight the need to focus on social and relational communication. Future research should explore how effective such interventions are on social and participation outcomes and look at real conversations between friends, identifying what helps or hinders communication. It would also be valuable to conduct long‐term studies on how friendships change over time and include the perspectives of friends to get a fuller picture of how relationships evolve after a stroke.	8	Core
[42], the United States	12 participants (8 men and 4 women) with a TBI. The mean age was 24.8 years, and the time since injury ranged between 1.5 and 5 years. 50% of the sample was European‐American, while other race/ethnic groups represented included Latina/o (*n* = 2), Filipino (*n* = 2), African American (*n* = l) and Native American (*n* = l). All participants were students enrolled in a non‐credit, specialised brain injury support programme offered at a community college.	Discover how community college students with TBI engaged within the community college and beyond in terms of their lifestyles, goals, relationships and strategies.	Interviews analysed using a Modified Analytic Induction Approach guided by phenomenology.	None reported.	Three themes were identified: Change in lifestyle and goals (change in lifestyle; a new sense of purpose; goal realignment); Changes in social engagement (from popularity to obscurity; dating and relationships on hold; elusive friendships; barriers to participation); Helpful strategies (involvement in student groups; use of academic accommodations; helpful programmes).	None reported.	7	Central
[40], the United Kingdom	24 participants (15 men and 9 women) with dysarthria as a result of stroke. The age range was 34–86 years, and the time since onset ranged between 2 and 34 months. 1 participant lived in very high deprivation and 7 in very low deprivation. No further socio‐economic or ethnicity data reported. The same sample as [39] was used.	Investigate the beliefs and experiences of people with dysarthria as a result of stroke in relation to their speech disorder, and to explore the perceived physical, personal and psychosocial impacts of living with dysarthria.	Semi‐structured interviews analysed thematically adopting a constant comparative methodology. Philosophical orientation not stated.	None reported.	Four themes were identified, two related to social isolation and connection: Psychosocial consequences (changes in self‐identity; relationship‐based disruptions; social disruptions; emotional disruptions; stigmatisation/perceived stigmatisation); communication.	Reasons for the lack of linkage between the severity of speech disorder and psychosocial impact should be examined further. A quantitative investigation of the relationship between severity and psychosocial impact may generate insights into determining factors.	4	Peripheral
[23], Australia	23 participants (20 men and 3 women) who had sustained a severe TBI. The mean age was 36.96 years, and the participants were 2–20 years post‐injury. Participants were British (56%), Southern European (22%), Eastern European (13%) and Arab (9%). No socio‐economic information.	Understand the post‐injury experience of friendship from the perspective of adults with severe TBI.	In‐depth interviews analysed using constructivist grounded theory. Quantitative data was collected and reported separately.	General strategies to enhance communication (e.g., quiet, familiar environment with minimal distractions, use of simple language without complex grammatical structures, and breaks to counter fatigue) and specific scaffolding procedures to minimise the effects of the most frequently occurring cognitive‐communication deficits in this population were used. As much as possible, the specific scaffolding procedures were used as a last resort when the meaning of the participant's discourse was obscured or no longer related to the purpose of the interview.	The post‐injury experience of friendship was broadly conceptualised as ‘going downhill’ with four overlapping phases: losing contact, being misunderstood, wanting to share and hanging on.	Friends themselves and the nature of the connection will contribute to differences in outcome, and understanding these associations will help tailor interventions based on dyadic and group factors. Friendship development and maintenance are frequently underpinned by shared activity and experience and reinforced by concrete reminders of these times. Thus, research into the short‐ and long‐term outcomes of friendships built within shared activities is also warranted. Working with people who have positive friendship outcomes could also provide valuable insights into effective strategies. Authors also suggest consideration of the diverse ways in which people maintain or develop social connections and recognise the breadth of sources of connections beyond friendship. Given the findings about ‘being misunderstood’, they suggest a role for education of others and reducing stigma about ABI.	8	Core
[17], Australia	20 participants (16 men and 4 women) with a TBI. The mean age was 35.2 years and the time since injury varied between 5 and 20 years. Participants were British (12), Southern European (4), Eastern European (2), Arab (1) and Polynesian (1). 1 participant was employed, 3 were volunteers, 2 were in vocational training and 13 participated in group community‐based leisure activities with attendant care support.	Explore how adults who have sustained severe TBI conceptualise self and maintain social connections several years after injury.	In‐depth interviews analysed using a constructivist grounded theory approach.	General strategies to enhance communication (e.g., quiet, familiar environment with minimal distractions, use of simple language without complex grammatical structures, and breaks to counter fatigue) and specific scaffolding procedures to minimise the effects of the most frequently occurring cognitive‐communication deficits in this population were used.	Three themes were identified, one of which was related to social connection: Staying connected: Factors that create a Sense of Connection between Self and Society (family; friends; paid carers; pets; concrete reminders; the ‘self’ narrative).	Future research should directly explore the effectiveness of socially symbolic representations, or ‘social snacks’, in supporting individuals with TBI, who often report unmet belonging needs. Digital tools like electronic communication and photography offer ways to maintain social connections when direct interactions are limited. Direct exploration of the effectiveness of such tangible reminders of social connection in the context of TBI rehabilitation is also recommended.	9	Central
[43], Australia	7 participants (6 men and 1 woman) with post‐stroke aphasia. The age range was 43–93 years and the time since stroke varied from 4 to 30 months. No ethnicity or socio‐economic data reported.	Understand the lived experience of interpersonal relationships for people with aphasia over the first year following the onset of stroke.	Semi‐structured interviews analysed using a constant comparative method within a constructivist grounded theory approach.	A range of strategies were used to support participants to convey messages. These fell within a ‘total communication’ approach including the use of gesture, drawing and writing. Modifying questions, providing repetition and clarification of meaning were also used.	The progression of interpersonal relationships over time following the onset of aphasia has six themes: Seeking Refuge within the Inner Circle; The Inner Circle Changes; Adjustment within the Inner Circle; Stepping Outside of the Inner Circle; Changed Landscapes with Friends; Connecting with Friends. Early on, participants retreated into a core group of close others characterised as their ‘inner circle’. During the early stages, inner circle relationships were subject to challenges and changes as people learned to live with aphasia. As they felt ready, people with aphasia began to reconnect with friends. This process was often mediated by spouses and, in the early phases of recovery, was limited and challenging. Reconnection with friends brought with it varying degrees of connectedness; however, for those without strong inner circle relationships, marked isolation occurred at all points throughout the year following discharge.	Further research around these emerging constructs is needed in a more extensive and diverse group of participants with aphasia, including females, with cultural and geographical diversity and differing injury severity. Studies should also adopt a timeline of more than a year, as people continue a process of adjustment for many years post‐stroke. Research could also examine participants with different relationship statuses individually, including those who are married, divorced and widowed, to see if different perspectives emerge.	8	Core
[70], the United States	77 participants (all male veterans) who had experienced a stroke. The mean age was 66.21 years. 18 were Black, 29 Puerto Rican Hispanic and 30 Non‐Hispanic whites. No socio‐economic information was reported.	Understand what experiential characteristics contribute to connectedness or social isolation over the first year of stroke recovery.	Semi‐structured interviews were conducted at 1, 6, and 12 months post‐stroke in the stroke survivor's home (as part of a larger longitudinal study). These were analysed using a grounded theory approach. Philosophical approach not stated.	Interviews with Puerto Rican participants were conducted in Spanish.	Two themes were identified: Characteristics of connectedness (Availability of others; Support from others; Interaction with others and community; Ability to contribute; Ability to engage in intimate relations) and Characteristics of isolation (Unavailability of others; Lack of support; Isolation from others and community; Inability to contribute; Inability to engage in intimate relations).	Future research should focus on exploring stroke survivors' experiences of connectedness and assessing how interventions targeting this aspect, rather than just social support, can impact recovery. Additionally, studying the effects of addressing survivors' sense of isolation could lead to practical improvements in clinical practice and enhance the quality of life for stroke survivors.	6	Core
[73], Sweden	8 participants (6 men and 2 women) with a TBI. The age range was 29–53 years, and the time since injury varied between 7 and 15 years. 4 participants were employed. No further socio‐economic or ethnicity data reported.	Elucidate the meaning of feeling well for people with moderate or severe TBI.	Semi‐structured interviews interpreted using a phenomenological hermeneutic method.	Questions were designed to be concrete and direct, and the scheduling of the interview was flexible.	One theme was identified: The unfamiliar becomes familiar (finding strength; regaining control over everyday life; being close to someone; being good enough).	Further studies should be conducted to gain more knowledge about feeling well with TBI, for example, in people who have lived only a short time with the injury and in the family members of people with TBI.	9	Central
[44], the United States	16 participants (8 men and 8 women) with moderate‐to‐severe brain injuries. The mean age was 46, and participants sustained their injuries at least 6 months prior. 81.3% of participants were Black/African, 25% were Hispanic/Latin American, and most had a high school diploma or general education diploma (68.8%).	Describe the social support structures and experiences of people with TBI.	Semi‐structured interviews were conducted and structured around a social network mapping exercise. Thematic analysis was used to analyse data. Philosophical orientation not stated.	None reported.	Two themes were identified: Structure of social networks (social networks are made up of families; proximity influences the type of support) and quality of social support (commitment vs. indifference; doing things with and for others adds meaning; and ‘they just love me’).	Future research should explore how ADL support influences perceptions of social support and evaluate models of social support that may overemphasise instrumental and informational support. Additionally, studies should examine the experiences and perspectives of social support providers to further enhance understanding in this area.	9	Central
[45], Canada	6 participants (3 men and 3 women) who had a stroke. The age range was 40–68 years; participants were interviewed at 6, 9, 12, 18 and 24 months post‐stroke. 3 participants were retired, 2 on sick leave and 1 on long‐term disability leave. Ethnicity information not reported.	Obtain a deeper understanding of the process of re‐engagement in personally valued activities post‐stroke.	Interviews at 6, 9, 12, 18 and 24 months post‐stroke which were analysed using a constructivist grounded theory approach. Participants were recruited from a larger longitudinal study. This study was conducted within a constructivist paradigm.	None reported.	Two themes were identified: social connection and being in charge.	Research is needed to assess programme effectiveness and explore re‐engagement in valued activities for those with communication challenges or living in isolation.	9	Peripheral
[46], Ireland	12 participants (all women) who had experienced a mild‐to‐moderate stroke. The age range was 20–48 years and the time since stroke varied between 10 and 29 months. No ethnicity or socio‐economic data reported.	Explore the experience of stroke among young women in Ireland.	Semi‐structured interviews analysed using interpretative phenomenological analysis.	None reported.	Four themes were identified, two related to social connection: a desire for peer support and the impact of stroke on relationships.	The psychological impact of stroke on young men should be explored. Additionally, individuals with aphasia and other impairments that hinder interview participation were excluded. It would be valuable to investigate the experiences of younger stroke patients with post‐stroke aphasia by providing communication support.	8	Peripheral
[47], Canada	22 participants (15 men and 7 women) who had sustained a moderate or severe TBI. The mean age was 42.4; the mean time since injury was 12.8 years. Participants were Canadian: 86.4%, Haitian: 9.1% and Portuguese: 4.5%. Socio‐economic information not reported.	(1) to describe the social participation of persons 10 years or more after their head trauma; (2) to identify the personal and environmental factors that play key roles in their social participation; (3) to describe the long‐term impacts of TBIs on their family and friends; and (4) to identify how the health and social services network (associations and inter‐sectoral groups) is used in response to their changing needs.	Participants followed up from a previous study 10 years ago. Semi‐structured interviews analysed using a thematic content analysis within a social–relational framework. Philosophical orientation not stated. Family members also included in the project; data reported separately.	The interview guide was created following guidance from patient representatives in a focus group.	Eight determinants and barriers of social integration were identified: (Safeguarding/developing family life; Having a spiritual life; Receiving long‐term follow‐up services; Being unable to return to work; Having a depressive episode; Abusing alcohol or other illicit substances; Experiencing divorce or separation TBI sequelae).	A research programme should be established to document post‐rehabilitation interventions in public and community networks and that the workings of the health continuum, involving professionals in how post‐rehabilitation problems are addressed.	6	Peripheral
[48], Ireland	11 participants (9 men and 2 women) with an ABI. The mean age was 45.7 years and participants were 1.5–10 years post‐injury. All participants were service users of a voluntary organisation supporting people affected by ABI in Ireland. No ethnicity or socio‐economic information.	Explore the experience of loneliness in individuals with ABI.	Semi‐structured interviews analysed using thematic analysis. Philosophical orientation not stated.	Communication aids and time‐out breaks were used as needed.	There were three overarching themes in the healing process: Internal Loneliness (shattered to pieces, left behind, wishing for an open wound; papering over the cracks; rejecting parts of the self), Healing the Cracks (processing of emotions; psychoeducation; the pre‐ and post‐injury self being held in mind as acceptable, by the therapist; mindfulness; developing dialectical thinking), and Visible with Cracks.	Future research should examine the therapeutic processes and relationships that benefit individuals with brain injuries to help refine therapeutic interventions. Additionally, research should explore what motivates individuals to seek psychotherapeutic support, including the influence of family and friends in encouraging post‐injury healing and community engagement.	9	Central
[49], the United States	9 participants (5 men and 4 women) who had experienced a stroke. The mean age was 54.7 years and the time since injury varied between 2 and 29 years. No ethnicity or socio‐economic data reported.	Identify dimensions of QoL that are important to patients after stroke.	Focus group analysed using thematic analysis. Philosophical orientation not stated.	None reported.	Four themes were identified, one of which was related to social connection: social relationships (social support; communication; independence).	It is important to address the gap in understanding between medical staff and patients about what it ‘feels like’ to experience a stroke, as well as the differing perspectives of caregivers and patients. As QoL is a subjective phenomenon, it is critical that stroke QoL measures are informed by the subjective perspective of individuals who have had a stroke, making focus groups an ideal method for capturing this perspective.	7	Peripheral
[50], Ireland	14 participants (8 men and 6 women) with PWA. The mean age was 51 years, and participants were 14 months to 14 years post‐stroke onset. At the time of stroke, 12 participants were employed, but this had reduced to 2 at the time of data collection. Ethnicity not reported.	Explore the perspectives of working‐aged adults with post‐stroke aphasia in relation to social participation and living well with aphasia.	Semi‐structured interviews analysed following the principles of reflexive thematic analysis. The approach was framed by critical realism.	The topic guide, developed and piloted with PPI advisers, enabled semi‐structured interviews with flexible sequencing and open‐ended questions. Scaffolding, such as binary choices, yes/no questions and examples from earlier interviews, was used as needed. PPI contributors also helped ensure significant others did not ‘speak for’ participants with aphasia by addressing questions directly to participants by name and verifying all responses with them.	Social participation spanned five themes: Relationships and roles (accepted by others; loss of relationships and social roles; parenting changes and role reversal; new relationships); Social support (practical support and encouragement; mixed feelings about support); Peer network (somewhere to go is most important; others in the same boat; meeting other aphasia families; opportunities to contribute, participate; aphasia activism and politics).	Future research should explore how to better support flexible, lifelong social access and participation for younger PWA in real‐world settings. This includes examining how PWAs navigate various communication formats, such as social media, and exploring interventions such as targeted communication partner training, self‐management of communication needs and social media training to address subtle challenges such as friendship loss through ‘ghosting’.	9	Central
[10], New Zealand	5 participants (2 men and 3 women) with severe ABI. All participants were living in a residential care facility, and they were involved in active goal‐directed rehabilitation. The mean age was 54.2 years, and the time since injury varied between 1 and 36 years. No ethnicity or socio‐economic data reported.	Examine the views and experiences of people with severe acquired brain injury regarding the place of ‘life goals’ in residential rehabilitation.	Semi‐structured interviews analysed using Interpretative phenomenological analysis.	None reported.	Three themes were identified, one of which was related to social connection: Being ‘part of things’ (Intimate connectedness through family relationships; Relational connectedness through friendships; Collective connectedness through citizenship).	Future research should focus on developing theoretical frameworks and interventions that better support individuals with severe ABI in achieving life goals, particularly in terms of social relationships and identity. It is also essential to recognise that a person's environment can change after severe ABI, and those in residential care need enhanced support and creative solutions to achieve their goals.	9	peripheral
[51], Sweden	19 participants (10 men and 9 women) who had a stroke. The mean age was 74.5 years, and the time since stroke varied between 1 and 19 years. 8 Participants were retired, 3 on sick leave and 8 in employment. Ethnicity not reported.	Investigate the lived experience of resilience and participation and their relationship to quality of life after stroke in Sweden.	Semi‐structured telephone interviews analysed using content analysis. Philosophical orientation not stated.	The interview guide was piloted with one stroke survivor.	Five themes were identified, two related to social connection: Meaningful values in life and Support (The importance of family and closest friends) and treatment from social relations (Social relationships work well and have sometimes deepened; Social relationships have become complicated and how they were treated).	Future research could include an examination of the co‐creation of resilience, which would expand the concept beyond individual adjustment to encompass a partnership between individuals/family caregivers and broader developments at service system and societal levels, creating a more responsive milieu for people with stroke.	9	Central
[52], Canada	11 participants (5 men and 6 women) who had a stroke and attended BLAST (the community peer‐led group), 4 with aphasia. The age range was 41–83 years, and the time since stroke varied between 3 and 31 years. 6 participants were White, 3 Asian, 1 Hispanic and 1 Southeast Asian. No socio‐economic data.	Explore participants' experiences in a community‐based, peer‐led, peer‐support group for stroke survivors. Furthermore, the research examined why individuals choose to participate in the group, the key elements and strengths of the group, and possible areas for improvement.	Semi‐structured interviews were analysed using constructivist grounded theory following participation in a peer‐led, peer‐support group for people with stroke. Relativist ontology, constructivist epistemology.	The interview questions were pilot tested with people with lived experience, then modified in regard to the length and specificity of the questions. For participants with aphasia, interviewers provided increased length of time for responses, encouraged use of technology to support communication (e.g., type‐to‐voice communication and typing responses), and encouraged use of on‐site support people if available (e.g., friends or family). Applied recommendations from the literature on interviewing people with brain injury.	Three themes were identified, two of which were related to social connection: Buddies helping buddies (peers providing insight into recovery; sharing in peers' successes in gaining function) and creating authentic friendships (understanding the experience; laughing and being silly together).	Experimental research is needed to determine the efficacy of peer‐led, peer‐support groups on stroke recovery and community integration. Future policy research is needed to explore potential funding for peer‐led, peer‐support groups for stroke survivors. Finally, further qualitative research is needed to explore the experiences of attending peer‐led, peer‐support groups for other neurological conditions.	9	Central
[53], the United Kingdom	10 participants (5 men and 5 women) who were experiencing post‐stroke aphasia. The age range was 53–84. 5 participants were black, 4 white and 1 mixed race. No socio‐economic data reported.	Explore the views of people with aphasia and their significant others about peer‐befriending	Semi‐structured interviews analysed using Framework Analysis. These were conducted within a feasibility trial (SUPERB) on peer‐befriending for people with post‐stroke aphasia and low levels of distress. Philosophical orientation not stated.	Pictures and photographs were used to support communication, and ‘total communication’ techniques such as gesture, drawing, facial expression and tone of voice were used to support speech.	Participants identified factors about the peer‐befrienders which influenced their experience: experience of stroke and aphasia; befriender character traits and rapport; conversation topics and activities.	On occasion, a befriender but not their significant other was interviewed, or vice versa. Interviewing both individuals would have enabled further elucidation of differences between perceptions of the intervention between the two groups.	8	Peripheral
[72], Ireland	15 participants (10 men and 5 women) with an ABI. The mean age was 41.93 years, and the time since injury ranged between 4 and 18 years. No ethnicity or socio‐economic data reported.	Explore whether there is evidence of altered social identities and changes in associated social identity resources after ABI, and how these changes might contribute to rehabilitation and adjustment post‐injury.	Semi‐structured interviews analysed using thematic analysis, informed by a social identity approach. Philosophical orientation not stated.	Questions were designed to be easy to answer.	Four themes were identified: Changed Social Relationships: As soon as the words ‘brain damage’ are mentioned, people run away; Families as Identity Resources post‐ABI: They really did help bolster me up; Group activity as recovery post‐ injury: We kind of treat each other like as if we're at work; It's like getting reset: Changing views of the self as a post‐injury social identity process.	Examining social identity change quantitatively among those affected by traumatic events and injuries is an important avenue for future research. Future work could also usefully orient to the therapeutic value of social identity management of chronic conditions to support the rehabilitation and recovery in conditions such as ABI.	7	Central
[54], the United Kingdom	10 participants (4 men and 6 women) who had a stroke. The mean age was 48.8 years, and participants were 4–20 years post stroke. No ethnicity or socio‐economic information reported.	Explore the concerns, perspectives and experiences of survivors following a stroke	Semi‐structured interviews, five face‐to‐face and five via e‐mail. Data was analysed using Interpretative Phenomenological Analysis. Quantitative data was collected and reported separately.	E‐mail interviews were used as a means to include people who might not otherwise be willing to take part.	Four themes were identified, with two related to social connection: Gender, romance and sexuality and social interaction.	Current research and support emphasise caregiver burden but must consider the changed and often strained interpersonal relationships of family caregivers and stroke survivors.	7	Central
[55], Sweden	20 participants (14 men and 6 women) with aphasia, 18 who had experienced a stroke and 2 a TBI. The mean age was 57.5 years, and the time since injury varied between 3 and 11 years. 2 participants were working part‐time, 3 tried to work but could not, 1 was retired and 11 had a disability pension or were receiving sick pay. Ethnicity information not reported.	Describe aphasic individuals' experiences of everyday activities and social support in daily life.	Semi‐structured interviews analysed using qualitative content analysis. Philosophical orientation not stated.	If a participant had problems with explaining something during the interview, paper and pen were made available for them to draw pictures, or the interviewer repeated the question or tried to explain it in another way.	Three themes were identified, two of which were related to social connection: Social support in daily life (Social support at the onset of aphasia and for a long time after; The meaning of support from community service; Support from relatives and friends) and social life at present (Feeling lonely; Relationships with relatives and friends).	Further studies are needed to improve our knowledge of everyday activity and social support for people with aphasia, and what it means to live with aphasia.	7	Peripheral
[56], the United States	7 participants (5 men and 2 women) with TBI. The mean age was 37 years, and participants were 10 months to 22 years post‐injury. One participant identified as American Indian, while the remainder identified as Caucasian. No socio‐economic data reported.	Understand the social interactions of individuals with TBI by exploring how individuals with TBI describe their social interaction experiences.	Two semi‐structured interviews with each participant analysed using constructivist grounded theory.	The interviewer read aloud simply worded questions, encouraging participants to refer to the interview guide and offering repeats or clarifications as needed.	Five themes were identified: Social life; self‐views; family relationships; friendships; and rehabilitation goals.	Continued research into the long‐term needs of people with TBI, especially in the area of social interactions, is needed to determine if there is a requirement for long‐term support. Exploring the practical strategies for improving social connections, peer support and social engagement for individuals with TBI is also needed.	3	Central
[57], the United Kingdom	29 participants (17 men and 12 women) who had a stroke, 10 with aphasia. The mean age was 68 years, and the time since injury varied between 8 and 15 months. 15 participants were White British, 6 White non‐British, 6 Black and 2 Asian. Socio‐economic data not reported.	Explore why people lose contact with their friends, whether there are any protective factors and how friendship loss and change are perceived by the individual.	Semi‐structured interviews analysed using the ‘Framework’ method. Philosophical orientation not stated.	The interviewer took care to modify her own language and use and be responsive to any communication modality favoured by the participants, including writing, gesture, the use of objects in their home environment and communication aids such as a communication passport. The interviewer also verbally commented on the participants' nonverbal gestures as they occurred, which helped to ensure their meaning was correctly understood, and meant there was a verbal record on the audiotape to be analysed later. Finally, where a participant had aphasia, the interviewer brought along a booklet with the topics laid out in an aphasia‐friendly manner (e.g., key words emboldened, use of white space). This book was used flexibly, thus preserving the ability to cover topics as they came up, while still giving a level of ‘scaffolding’ to the interaction to aid comprehension.	Four themes were identified: What are the causes of friendship loss after a stroke? (loss of shared activities; reduced energy levels; poor mobility and other physical symptoms; unhelpful responses of others; environmental barriers; aphasia and changing social desires of participants; ‘I seem to be closing in on myself’), What factors help to protect friendships? (Feeling ‘close’ to a friend; Distance; Availability of the friend; Not activity‐based before the stroke; Regular, supportive groups; Family friends; Having a ‘friends‐based’ social network before the stroke) How is friendship loss and change perceived by the individual? and unpacking the relationship between depression and loss of friends.	Follow how friendship patterns develop in the longer term after a stroke, unpicking the potential benefits of forming new friendships and acquaintances. Further investigation could also explore friendship change and loss from the perspective of the friend, particularly for those with aphasia. Finally, research could explore more fully how young stroke survivors perceive friendship change.	8	Central
[32], the United Kingdom	29 participants (17 men and 12 women) who had a stroke. The mean age was 68 years, and the time since injury varied between 8 and 15 months. 21 participants were White, 6 Black and 2 Asian. No socio‐economic data.	Explore whether there are predictable patterns in which support functions are provided by particular network members, how receiving support is perceived and what support functions are most valued.	Semi‐structured interviews were conducted and analysed using the framework method. Philosophical orientation not stated. This study was part of a larger project exploring quality of life and social support post‐stroke.	Total communication (participants were encouraged to use all communication modalities to get across their point), allowing additional time and scaffolding was used.	Two themes were identified: Support provided by different network members (Spouse; children; relatives; friends); What support functions are most valued post‐stroke? (emotional support; social companionship; ‘responsive’ tangible support; informational support; manner, context and purpose of functional support)	Research should seek sensitive ways to measure the sense of feeling connected to others. Future measures should consider further how best to measure the context (e.g., contribution, reciprocity and quality of relationship) and manner (e.g., responsiveness and sensitivity) of support, as well as the value of everyday companionship, following a stroke.	8	Central
[58], Denmark	11 participants (7 men and 4 women) who had a stroke. The age range was 25–66, and interviews were conducted at 3 and 12 months after stroke. 6 participants lived in Denmark and 5 in Norway. 5 participants were retired, 5 in work‐training and 1 in full‐time employment.	Explore the recovery trajectory experiences of stroke survivors as they unfold in the Danish and Norwegian contexts.	Two semi‐structured interviews analysed using phenomenological analysis.	None reported.	Four themes were identified, two of which were related to social connection: The involvement of family, social network and peers (Help from family members; Significance of family support; Conversations about the stroke and the new life; Comparing with others and interplay with other peers) and social structures that limit the recovery process (Return to work—a particular issue in the recovery process; Getting out and about—transport and driving ban limiting recovery).	Future research should develop and assess the effectiveness of a learning approach in rehabilitation. The next step in a Norwegian‐Danish collaboration could be to develop and evaluate a return to work programme based on these principles.	8	Peripheral
[59], the United Kingdom	11 participants (9 men and 2 women) with TBI. The mean age was 49 years, and participants were 5–33 years post‐injury. All participants attended the Head Forward Centre, a social rehabilitation day programme. 1 participant was a volunteer, and the rest were unemployed. Ethnicity not reported.	Explore TBI survivors' subjective account of the challenges encountered in sustaining friendships, as well as gaining insight into their understanding of such difficulties.	Semi‐structured interviews analysed using thematic analysis. Philosophical orientation not stated.	Questions were offered to participants in a stepwise fashion, from general to more specific, to facilitate the recollection and organisation of their ideas. A collaborative interview approach, where the interviewer can have an active role scaffolding the generation of narratives, was adopted.	Four main themes were identified: The impact of long‐term cognitive and behavioural problems on relationships; Loss of old friends; Difficulties making new friends and relating to other survivors to fight social isolation (sameness).	Future research should explore whether the emphasis on ‘sameness’ in friendships among TBI survivors is unique to those in therapeutic communities, like the Head Forward Centre used in this study, or a broader experience among survivors outside such environments. It could be useful to investigate the role of sameness in survivors who attend ‘drop‐in’ support groups, where informal social interaction occurs but is not therapeutically planned.	6	Core
[34], Australia	39 participants (23 men and 16 women) who experienced an ABI (54% stroke). The mean age was 52 years, and the time since injury varied between 8 months and 46 years. No ethnicity or socio‐economic data reported.	Evaluate the Valued Living After Neurological Trauma (VaLiANT) intervention to complement and aid in the interpretation of the main trial results and to assist in the refinement of the intervention for future delivery and evaluation.	Semi‐structured interviews analysed using reflexive thematic analysis, following participation in the VaLiANT intervention. A critical realist orientation to the data was adopted.	None reported.	Three themes were identified, one of which was related to social connection: The value of connection and belonging.	A qualitative metasynthesis could help map these findings onto existing adjustment and well‐being models, guiding future intervention development and improving outcome measurement. Additionally, many positive outcomes reported from the intervention were subtle and varied among individuals, which may not be captured by standard outcome questionnaires. Qualitative methods can provide valuable insights by highlighting the nuanced effects of complex, holistic interventions.	8	Peripheral
[60], the United States	6 participants (4 men and 2 women) who had a stroke. All participants were living in Appalachia. The mean age was 47 years. No ethnicity or socio‐economic data reported.	Describe the experiences of adults who had survived ischaemic stroke and who were living in Appalachia.	Semi‐structured interviews analysed thematically. Philosophical orientation not stated.	None reported.	Five themes were identified, one related to social isolation and connection: Negative emotions, including anger, guilt, loneliness and depression.	Home‐based interventions need to be developed which lessen frustration, enhance connectedness, improve access to support, increase home‐based services, and work with patients to enhance characteristics that enable a person to ‘step forward’ after stroke.	8	Peripheral
[61], Sweden	11 participants (5 men and 6 women) who had experienced a stroke. The mean age was 58 years, and the time since stroke varied between 2 and 14 years. 9 were born in Sweden and 2 elsewhere. 2 were retired, 4 receiving sick compensation, 1 on sick leave and 4 working full‐time.	Understand life after stroke through the lens of participants' cameras, and hence their views and experiences guided this study.	Photovoice, an action research method, was used. Participants photographed in everyday life for up to 4 weeks and then met to discuss all images in a focus group setting. These were analysed using a thematic analysis. Philosophical orientation not stated.	Photos were used to generate discussion during focus groups.	Five themes were identified, two of which were related to social connection: A driving force to participate in society and finding meaningful relationships in daily life.	To investigate the role of specialists in facilitating long‐term adaptation processes after stroke.	8	Peripheral
[62], Sweden	11 participants (7 men and 4 women) who had a stroke. The mean age was 48 years, and the time since injury was 7–8 years. 8 participants were working full‐time,1 working part‐time, 1 unemployed and 1 on parental leave. Ethnicity not reported.	Enhance the understanding of long‐term participation in working‐aged people 7–8 years after stroke.	Semi‐structured interviews analysed using thematic analysis. Philosophical approach not stated.	The interview guide was created following guidance from patient representatives and was pilot tested on two individuals with long‐term stroke.	Four themes were identified, one of which was related to social connection: Social life 7–8 years after stroke.	It is still not known exactly what aspects are important for facilitating the process of managing successful participation. More knowledge about coping strategies that improve participation in activities many years after a stroke is needed, so that more people can reach a state of positive identity and participation.	8	Peripheral
[31], Australia	13 participants (6 men and 7 women) with an ABI. The mean age was 36.9 years, and the mean time since discharge from hospital post injury was 15.2 months. No ethnicity or socio‐economic data reported.	Explore the transition experiences from hospital to home of a purposive sample of individuals with ABI.	Semi‐structured interviews analysed inductively following a phenomenological approach.	None reported.	Eight themes were identified, two related to social connection and isolation: the role of family caregivers; friendship networks and community involvement.	Further research is necessary to conceptualise the transition phase within the rehabilitation continuum. Prospective studies that explore the experiences of individuals with ABI and their caregivers at specific time points during the transition phase would enable a more in‐depth understanding of the process and facilitate the identification of factors predictive of poor psychosocial outcomes.	9	Peripheral
[63], Sweden	16 participants (4 men and 12 women) who had experienced an aneurysmal subarachnoid haemorrhage. The age range was 30–74, and the time since injury was 1 year. 5 participants were employed, 5 retired and 6 on sick leave. Ethnicity not reported.	Describe perceived support, support needs and self‐care among individuals during the first year after an aneurysmal subarachnoid haemorrhage.	Semi‐structured interviews analysed using content analysis. Data was also collected from caregivers. Philosophical orientation not stated.	None reported.	Two themes were identified, one related to social connection: Social support (including esteem/emotional support, informational support, social companionship and instrumental support).	None reported.	8	Peripheral
[64], the United Kingdom	29 participants (16 men and 13 women) who had a stroke. The mean age was 61.9 years. 4 participants were employed, 3 unemployed, 13 retired, 5 medically retired and 4 on sick leave. No ethnicity data.	Explore and discover the meaning of loneliness specific to stroke survivors in Northeast England.	Semi‐structured interviews analysed using the framework method. Philosophical orientation not stated.	None reported.	Five themes were identified, two related to social connection and isolation: Loneliness is being alone for a long time; Stroke, social relations and loneliness.	A new scale, particularly for stroke survivors to measure their experiences of loneliness more accurately, should be constructed. This should contain items about how they identify themselves; for example, whether they can continue to do things they used to do, and how much independence they have lost since the stroke. It should also capture stroke survivors' communication with others, including both the perception of understanding by others, and the stroke survivor's confidence in expressing themselves in front of others.	7	Peripheral

### 2.6 Quality Assessment

Quality assessment occurred in the broader context of appraising the contribution of each paper to the review, as outlined in Whiffin et al. [65]. Papers were assessed on their rigour, relevance and resonance. Rigour was evaluated through the standardised critical appraisal instrument from JBI for qualitative research [66], relevance was based on the research question and study participants, and resonance on the content, style and scope of the results. Each paper was classified as ‘Core’, ‘Central’ or ‘Peripheral’, reflecting these elements. Full appraisal details are provided in Supporting File 2. J.A. assessed all papers, while S.H. and F.G. independently evaluated half to ensure consistency. Disagreements (*n* = 3) were resolved through a single discussion. Data extraction and synthesis were conducted for all studies, irrespective of their methodological quality.

### 2.7 Synthesis Methodology

The thematic synthesis framework [67] was used to summarise and synthesise the qualitative data. This was approached from a critical realist perspective, which acknowledges that individuals' experiences are shaped by their subjective perceptions and underlying social structures and mechanisms [68].

### 2.8 Thematic Synthesis Process

J.A. read the results of the included papers several times, noting any reflections. Papers were then coded line‐by‐line semantically and latently using NVivo. This process started with the ‘core’ papers, and the codes developed were then applied to ‘central’ and ‘peripheral’ papers, where few new codes were identified, indicating that ideas had been thoroughly represented [65]. The codes were repeatedly refined to form higher‐order descriptive themes. The interpretations of these were discussed, leading to the inductive generation of analytical themes. The research team included two PhD students, a clinical neuropsychologist turned academic with 25 years of experience, a community health expert with 30 years of experience, and a patient and public involvement (PPI) collaborator whose husband experienced an ABI, who works professionally in the disability sector. Together, they brought diverse clinical, research and qualitative expertise, alongside a shared commitment to inclusive, community‐engaged research. These varied perspectives informed the thematic focus, ensuring sensitivity to participant experiences through reflexive consideration of potential interpretative influences. Sense‐checking was carried out with the PPI member to ensure that the interpretation was meaningful.

### 2.9 Trustworthiness

Trustworthiness in qualitative research involves credibility, dependability, transferability and confirmability [69]. Credibility was enhanced by including a large sample, critical reflexivity and sense‐checking interpretations. Dependability was supported by the robust systematic review process, audit trails and iterative theme refinement. Including rich participant quotes and contextual details supported transferability. Confirmability was established by grounding findings in data and transparently reporting decisions.

## 3 Results

### 3.1 Study Characteristics

The characteristics of the 37 included studies are presented in Table 1. Together, these captured the perspectives of 629 participants, comprising 396 men and 233 women. Ages ranged from 18 to 93 years, and the time since injury varied from 6 months to 46 years. The studies encompassed various ABI types, with some focusing on stroke (*n* = 22) or TBI (*n* = 7), and others including multiple forms of ABI (*n* = 8). Several focused on aphasia (*n* = 8) and dysarthria (*n* = 2). Brady et al. [39] and Dickson et al. [40] used the same sample but reported different data, so both were included. Three studies explored social connection and isolation within intervention contexts. Participant quotes are labelled accordingly.

Papers were dated from 2007 to 2024 and were conducted in the United Kingdom (*n* = 8), the United States (*n* = 7), Australia (*n* = 6), Sweden (*n* = 6), Ireland (*n* = 5), Canada (*n* = 3), Denmark (*n* = 1) and New Zealand (*n* = 1). Individual interviews (*n* = 35) were the primary method, with two studies using focus groups. Six papers were categorised as core, 15 as central and 16 as peripheral.

### 3.2 Key Themes

Three main themes were identified to describe the experience of social isolation and connection post‐ABI: (1) Navigating isolation: loss, communication and stigma, (2) The bonds that heal, and (3) Reconstructing the self and values. A summary is presented in Table 2.

**Table 2:** Summary of the themes identified.

Themes	Sub‐Themes
Navigating isolation: loss, communication and stigma	Social loss and the lived experience of isolation The weight of words Stigma, misunderstandings and the challenge of inclusion
The bonds that heal	The role of family and pets in connection Building connections through shared activities and reciprocity The power of peers and shared experiences
Reconstructing the self and values	Reconstructing identity Rediscovering personal values

These themes reflect a connected process: navigating isolation reveals challenges such as stigma, while meaningful connection reduces isolation and supports identity and value reconstruction, which further encourages connection. Table 3 demonstrates which studies contributed to each theme.

**Table 3:** Overview of study contributions to themes and sub‐themes.

	Navigating isolation: loss, communication and stigma			The bonds that heal			Reconstructing the self and values	
Study (author, year)	Social loss and the lived experience of isolation	The weight of words	Stigma, misunderstandings and the challenge of inclusion	The role of family and pets in connection	Building connections through shared activities and reciprocity	The power of peers and shared experiences	Reconstructing identity	Rediscovering personal values
[38]	Y	Y	Y				Y	Y
[71]	Y	Y	Y		Y			
[39]	Y	Y	Y				Y	
[41]	Y	Y	Y		Y	Y		
[42]	Y	Y	Y		Y			Y
[40]		Y	Y					
[23]	Y		Y		Y			Y
[17]		Y		Y	Y			
[43]	Y	Y	Y	Y	Y			
[70]	Y		Y	Y	Y			
[73]			Y	Y	Y	Y		
[44]	Y	Y	Y	Y	Y			
[45]				Y	Y			
[46]	Y		Y			Y		
[47]	Y			Y				Y
[48]	Y	Y	Y		Y		Y	
[49]	Y	Y	Y	Y				
[50]	Y		Y	Y		Y		
[10]	Y			Y	Y			
[51]		Y		Y	Y			
[52]		Y			Y	Y		
[53]			Y			Y		
[72]	Y	Y	Y	Y	Y			
[54]	Y		Y			Y	Y	
[55]	Y	Y	Y	Y				
[56]	Y		Y	Y				Y
[57]	Y	Y	Y		Y		Y	
[32]	Y	Y	Y	Y	Y			
[58]	Y	Y		Y		Y		Y
[59]	Y		Y			Y		
[34]			Y			Y		
[60]	Y			Y		Y		
[61]	Y	Y	Y	Y				
[62]	Y	Y	Y		Y			Y
[31]	Y		Y					
[63]		Y	Y					Y
[64]	Y	Y	Y	Y				

#### 3.2.1 Navigating Isolation: Loss, Communication and Stigma

The experience of isolation was identified as a pervasive theme, characterised by loss, communication difficulties, stigma and misunderstandings.

##### 3.2.1.1 Social Loss and the Lived Experience of Isolation

Social isolation was both a personal and relational experience characterised by the loss of friendships and support networks and negative changes within relationships. Whilst initial gestures of support from friends post‐injury were noted, individuals recounted the painful realisation that friendships faded with time, ‘*All of our friends sort of disappeared. They didn't stick around…. I don't think they could handle it*’ [31]. The loss of friends was particularly salient when individuals felt ignored or forgotten, ‘*I hated being ghosted … you sent a group text … and you don't get any response*’ [50].

Feelings of isolation persisted even when individuals were physically present with others due to a sense of disconnection, ‘*I can still be with people but feel lonely because I'm not the same as them. Like they can be doing things that I can't, and that makes me feel lonely*’ [64]. The opportunity to engage socially also diminished, as activities that once brought joy and connection became inaccessible, ‘*I can't go out like I did…. Normally, I would be out there to be with people and be out dancing. But there aren't that many things I can do anymore’*. A lack of encouragement from others increased isolation, ‘*No one says like, why don't you come out and try—they don't even question me*’ [44].

A sense of isolation often began upon returning home due to the stark contrast between the intensive support received at the hospital and the lack of care after discharge. This shift led to feelings of abandonment, ‘*Well, in the hospital I had attention. Here now, I have none…*’ [70]. Even when state support was available, many were dissatisfied with its quality, ‘*They do not help me with anything…*’ [55]. While family support was crucial to fill the gaps in formal support, not everyone had this available, compounding feelings of abandonment and isolation, ‘*I'm alone, no one takes care of me… I have family but they all have their own things to do*’ [70].

##### 3.2.1.2 The Weight of Words

Communication difficulties in those experiencing aphasia perpetuated isolation by impeding the ability to engage in meaningful interactions or maintain relationships. Participants mentioned that their ‘*circle of friends reduced dramatically’* [38], as others were unable or unwilling to adapt to their speech difficulties. Among those who remained, conversations tended to be minimal, ‘*Even if it's some friends coming here. You don't know how to…. I mean they're coming and say, hello…. But we don't—[talk and have the same conversations]’* [41]. This caused feelings of alienation, despite the presence of others, ‘*but even if I had a house full of people, I could still feel lonely and they could be all engaging with you but I just drift off and really you've no idea what's going on in my head’* [48].

A struggle was noted between the desire to be understood and have close relationships, while simultaneously feeling unable or unwilling to explain one's thoughts. These challenges and the frustration of not being able to ‘*get across what you want to say’* [51] caused some individuals to withdraw emotionally, ‘*right now there's a wall around me completely’* [38]. This also led to an avoidance of social situations, ‘*I don't want to go down to the pub because of it’,* and reduced engagement with others, ‘*I just say what's necessary and that's it’* [39]. These difficulties also made forming new relationships challenging, ‘*Because if you can't talk and get across what you want to say and feelings and everything like that, then … it means that you have a hard time making new contacts of a meaningful nature’* [51].

Supportive communication and alternative forms of interaction were crucial in combating isolation. Some participants expressed that verbal communication was not essential in close relationships, with silent understanding sustaining these bonds, *‘…he would sit there and stay with me for more than an hour and—and I wasn't able to say a word—you know. But then he's such a good friend’* [41]. In the friendships that persisted, supportive communication was a key factor. Friends were highly valued for their ability to ‘*not finish my sentences’, ‘slow down’, ‘allow time’* and ‘*be patient’* [71].

Text‐based communication was also beneficial, ‘*…I write better than I then I speak, so … we go on WhatsApp or I message’.* Tools like predictive text and AutoCorrect reduce cognitive load and support creative expression, ‘*GIFs and emojis … are my new love language’* [71]. Technology can also help to maintain long‐distance relationships, ‘*My daughter lives in California with my sister and brother. I get to talk to them about every other day’* [44]. However, it does not always foster meaningful interactions. Social media was often seen as superficial, ‘*They try to make contact but it's like “via Facebook” and that is about it. They don't try to make real contact with me; they don't call my cell phone or anything ever, probably because they don't want to know how I'm doing’* [42].

##### 3.2.1.3 Stigma, Misunderstandings and the Challenge of Inclusion

The invisible nature of an ABI and the lack of public awareness cause dismissive attitudes and a lack of understanding, creating barriers to connection. For instance, some members of the public mistakenly judge those with an ABI negatively and subsequently deny them access to resources, ‘*You cannot come on this bus, you are pissed’* [54]. Even professionals have similar judgements, ‘*The doctor thought I was drunk’* [39], highlighting the widespread nature of this issue. As a result, many individuals wished for a visible injury, ‘*I wished I had a broken leg, you can see it is a broken leg and you wouldn't say “oh no you haven't broken your leg”, you accept the person has a broken leg. People would relate to you with a broken leg’* [54].

The lack of understanding extended beyond strangers to friends and family. Several participants described being overlooked in conversations, with questions directed to others rather than them, ‘*Some people speak to Gillian [partner], asking how I am instead of asking me myself’* [39]. Many participants felt that others held negative assumptions about ABIs, which acted as a barrier to connection, ‘*A lot of friends, I found that as soon as the words “brain damage” are mentioned, people run away…. They are scared you are going to sit in a corner and dribble at them’* [72]. Similarly, another participant explained how others were ‘*dismissive because I'm a bit different’* and that ‘*most people can't understand me—they won't associate with me’* [23. These assumptions created feelings of inadequacy, ‘*It makes you feel a bit stupid’* [39].

Internalised stigma caused some individuals to put on a facade around others to avoid judgement, which had significant physical and emotional impacts, *‘When I'm mixing with other people I'm always trying to be the person I was. It's tiring…’* [59]. This can cause individuals to avoid social situations, exacerbating isolation.

#### 3.2.2 The Bonds That Heal

Meaningful connections with others help mitigate feelings of isolation by offering support, understanding and providing meaning.

##### 3.2.2.1 The Role of Family and Pets in Connection

Family members were highly valued for their unwavering support, which provided stability and reassurance. Relatives frequently ‘*rallied round’* [32] to offer emotional and practical support. Whilst friendships felt more volatile and fragile, family provided a sense of security and comfort, ‘*They're just here, and I know they're for me’* [17].

Family members were also appreciated for offering a safe space to vent, and they were typically more proactive in making contact, adapting to an individual's needs and providing practical support to engage in social interactions. A common theme throughout many of the included studies was gratitude for family and a deeper connection than before. One individual expressed, ‘*She helped me put my life back together … and she is still helping me today’* [44]. When support from family did encompass the above, participants explained not needing to rely on other connections, ‘*I have had support from my husband, I must say. And so it's probably the support that maybe—I may not have felt it, or not needed any other support, you could say, from outside, of others’* [51].

Pets were also a source of emotional support, ‘*I'm never lonely because I have Bess—she is who I confide in’* [17] and unconditional love, ‘*She is both company and I feel that I'm needed, that I'm loved…’* [73]. Beyond companionship, pets also facilitated social interaction, ‘*I go on walks with my dog and I'm able to have social interaction with other people…’* [60].

##### 3.2.2.2 Building Connections Through Shared Activities and Reciprocity

Participating in groups and hobbies provided an opportunity to build meaningful connections and reinforce bonds with existing connections. For example, going to the Legion (a charity‐funded social club) every couple of weeks was valuable for one individual, ‘*[I have] friends to meet there and (…) [it's] a place to get out and get to meet new people and old people’.* Similarly, another participant described the benefits of golfing, ‘*It's a very social sport, you know, and the girls that I golf with are a lot of fun (…) a good group of nice friends’* [45]. The shared enjoyment and camaraderie of a sport or joint interest can strengthen one's sense of belonging and provide purpose.

Helping others provided purpose and fulfilment and fostered social connection by enabling meaningful engagement, reducing isolation and promoting a sense of community, ‘*ya like a purpose or something, it's like after my brain injury I felt no purpose, no use, and that was a depressing lonely place to be but to be able to feel like you're helping other people to cope with or … something helping people who need it, that gives me a sense of happiness and takes away being lonely and depressed’* [48]. Meanwhile, reciprocity promoted a sense of mutual inclusion and reinforced connection, ‘*We help each other … he would call, I was calling. I would really sort of talk to him and he would really like talk to me and it was a good, good feeling’* [71].

##### 3.2.2.3 The Power of Peers and Shared Experiences

Peer interactions created safe spaces where individuals feel understood and accepted, which reduces isolation. One participant described how peer groups enable authentic connection, ‘*There's all sorts of people and they have all sorts of symptoms. But they are in the same boat … there is nothing that can't be talked about … you talk to people that know what you're talking about’* [50]. These interactions are marked by mutual understanding, ‘*It's just nice to talk with people who … knows what it's all about’* [41] and shared experiences, ‘*I can understand them [people with TBI] better but I can also relate to them like they can relate to me. Because they've all had the same problems, I've had the same problems, so it lets you relate and talk to each other about the same problems’* [59]. The absence of judgement creates a relaxed environment, which is enjoyed no matter the activity, encouraging conversations about feelings which can be otherwise challenging, ‘*we could play games, we could just sit and talk, we could watch television together, we could do whatever (…) It provided the opportunity to have some good chats about: “It's not going so well,” and about lots of things’* [58].

Discussions with peers further along in their recovery journey provided hope and encouragement, ‘*We'll do what we call a “Go Around,” where each person will revisit their journey. It is beneficial for new people coming in to hear somebody else's story and their progression … they get to hear from people who have had a stroke that it's not a sprint, it's a marathon, but you will get better’* [52] (intervention study). Peers can also give advice regarding practical strategies to implement, further encouraging recovery, ‘*I was impressed with the strategies that she used. Listening to her talk about them week after week, I started using them and they were helpful’* [34] (intervention study). These conversations also normalise difficulties and foster humour, ‘*It—it doesn't matter whether you make mistakes and all that because we—it— we—we're all—we're all—uh—not—not word‐perfect. Uh—anyway we—we laugh. And it—it—it's just fun’* [41].

#### 3.2.3 Reconstructing the Self and Values

Meaningful social connections provide essential interaction and support, fostering a sense of belonging and purpose, which facilitates identity and value reconstruction.

##### 3.2.3.1 Reconstructing Identity

Cognitive, emotional and communicative changes profoundly affect an individual's reality and sense of self post‐ABI. These changes can lead to social challenges, resulting in frustration, anger and self‐imposed isolation. Reconstructing one's identity is a complex but essential journey to overcome these negative feelings. One participant described the process as similar to grief, ‘*I really felt about it was that it's like a death, while no one has died I appreciate, but the old you is gone, you're going through a grieving process whether you know it or not, but grieving alone’* [48]. The loss of identity was particularly challenging, ‘*So when you go into the … post office … and people go “There's Bill. Bill had a stroke.” Everybody in the town knows, but it's still you, but it's not you … I should be “Bill from the bowling club” or whatever, not “Bill that's had the stroke”’* [39]. The loss of identity is closely linked to a disruption in familial roles, which was especially frustrating when it challenged traditional gender roles, ‘*I get very angry when I cannot do the things that I should be able to do. That my wife has to have so much of my care when I should be taking care of her’* (Murray and Harrison et al. 2004).

Despite these challenges, rebuilding a meaningful identity is possible. Forming meaningful connections, having positive interactions and engaging in shared activities and reciprocity play pivotal roles in this journey. As one individual noted, ‘*It was the making of me, it is making me stronger’* [38].

##### 3.2.3.2 Rediscovering Personal Values

An ABI can prompt individuals to actively (re)discover their values and priorities, uncovering genuine friendships and supportive networks that help alleviate isolation through meaningful connections. However, the realisation that old friendships no longer align with their current values can be isolating, ‘*I have completely made a distance from my friends, like my friends from high school, I'm into the social networks, and I see them on Facebook they are always drinking and partying, (what a waste), but I just don't see them in person…’* [42]. This led to a desire for meaningful, rather than superficial connections, ‘*a lot of people's interests are very shallow. Now it's like (things) don't matter so I guess I don't really have an interest in friends that are shallow like that’* [56].

Despite experiencing losses, individuals often found a deeper appreciation for the relationships that remained, ‘*You learn very well who your real friends are. Who likes you for who you are, and not because it was fun to have a party buddy. Those who were real friends are still there’* [63]. Rediscovery also included reconnecting with others, ‘*I try to pay as much attention to them as possible … before I didn't really call my mom and I went like 6 years without talking to my sister…’* [56] and positive changes to oneself, including being more considerate to others, ‘*You'd just be more aware maybe of what's happening around you … like I can't just ignore those kinds of things anymore*’ [38]. These changes foster stronger connections and greater engagement, creating more opportunities for connection.

## 4 Discussion

Social isolation was discerned as a pervasive feeling of loss driven by ABI‐related impairments, communication challenges and stigma. This review also identified key elements of social connection: understanding, acceptance, reciprocity, shared experiences and emotional support. These qualities foster feelings of belonging, security and purpose, encouraging a reconstruction of personal values and identity. This discussion explores how these findings enhance our understanding, with a focus on valued outcomes and contextual factors.

Social isolation can be seen as a direct consequence of an ABI and influenced by deeper social and environmental mechanisms. These include broader societal and systemic factors, such as stigma, that shape individuals' experiences. Individual factors, including cognitive, communicational, functional and physical impairments, contribute to isolation as they can increase the difficulty of connecting with others and maintaining relationships [74, 75]. Unlike other conditions with common physical impairments, including Parkinson's disease [76] or multiple sclerosis [77], ABI survivors face unique difficulties due to the dual challenges of invisibility and stigma. This includes misconceptions about their condition, such as being intoxicated [54], and assumptions regarding diminished intelligence [78]. Research shows that public attitudes are more negative towards ABI survivors than those with other conditions, which, when combined with low public awareness and inadequate rehabilitation resources, exacerbates feelings of isolation as they limit opportunities for meaningful connection and reinforce exclusion [79]. These factors align with Hammel et al.'s [80] framework, which highlights how social support and attitudes, systems and policies, and economic factors critically impact participation for people with disabilities. The interplay between environmental factors and ABI‐related impairments is overlooked in current research. Attending to this would help elucidate the key mechanisms driving isolation, allowing for the development or refinement of outcome measures and interventions that better reflect lived experiences.

It is well established that social connection is beneficial for emotional well‐being and combating isolation [17]. This review advances understanding by emphasising the importance of the quality of social connections rather than their presence alone. Key components of valued relationships include acceptance, emotional support and mutual understanding. These relationships are often built on reciprocity and shared activities, characterised by supportive and open communication. Such connections foster a sense of belonging and purpose [44] and accommodate an individual's evolving identity and personal growth [38]. As discussed, post‐ABI, individuals may experience positive shifts in life meaning, as represented in theories such as post‐traumatic growth [81]. This growth was evident in the rediscovery of participants' identity and personal values, supported by relationships that embodied valued qualities and created a safe, empowering environment [38]. This supportive atmosphere may promote valued living, where an individual acts in concordance with their values to enhance well‐being, highlighting how these relational components align with broader understandings of post‐injury well‐being [82, 83]. This may create a reinforcing cycle whereby meaningful social connections foster belonging, purpose and security, leading to more social interactions [43] and engagement in activities [84]. These promote post‐traumatic growth and valued living, which encourage further meaningful connections. This demonstrates how high‐quality connections are key to navigating the challenging dynamics post‐ABI and highlights the need for outcome measures that capture these nuances.

This metasynthesis demonstrated that different types of connections serve distinct purposes. Whilst family relationships typically provide vital emotional and practical support, including offering a safe space, maintaining contact and facilitating interactions, friendships can be more susceptible to loss due to communication difficulties and stigma [17]. Peer relationships were valuable for practical recovery support [85] and for fostering belonging, understanding, normalcy and resilience [7, 86]. While high‐quality relationships can mitigate the impact of stigma and exclusion, contextual constraints and enablers mediate individual experiences of connection. For instance, the capacity of family members to provide consistent support is influenced by caregiving resources and financial pressures [87]. When family members cannot provide support due to such factors, the experience of isolation is compounded [70]. Within the included studies, accounts of positive social connections and identity reconstruction were most prevalent in those engaged in structured community support [48, 59], employment, volunteering [17, 73], or peer‐support groups [52, 58]. However, these opportunities may not be available or accessible to everyone, potentially exacerbating inequalities. Inconsistent reporting of socio‐economic, demographic and other contextual data in the included studies limits the understanding of their role in social connection and isolation, which should be addressed in future research.

### 4.1 Limitations

The studies included in this review exhibit several limitations. There was limited use and reporting of PPI, limiting the relevance and acceptability of the findings [88]. Additionally, many studies failed to outline method adaptations for ABI accessibility, while those that did relied heavily on supportive communication in traditional interviews. To elicit richer, more nuanced insights and enhance accessibility, utilising creative methodologies is essential [89]. The diversity of participants was also limited. Due to differing structural barriers, different groups likely have unique experiences of connection and isolation, which were not captured. For instance, those living in rural areas often have less access to formal support [90], so they rely more heavily on informal networks. Lastly, approximately one‐third of studies did not specify their philosophical approach, raising concerns about the rigour and coherence of the findings. These limitations raise uncertainty about the completeness of the present findings. Within the review, PPI was primarily with a family carer, not individuals with ABI, which is acknowledged as a limitation.

### 4.2 Implications

This review demonstrates that social connection and isolation are multi‐dimensional, context‐dependent outcomes that are distinct yet interconnected. For instance, while our findings positioned internalised stigma within the context of navigating isolation to emphasise its immediate impact on social withdrawal, it also plays a role in identity reconstruction. Stigma may contribute to difficulties in letting go of a pre‐injury identity, or in cultivating a renewed, positive sense of self. Previous work has shown that maintaining social group memberships, a key component of identity continuity, is associated with higher levels of well‐being post injury [91]. This suggests that disruptions to identity, whether through stigma or other barriers, may have longer‐term implications for recovery and connection. However, understanding and acceptance from others may mitigate the effects of stigma and promote a sense of belonging and connection [38]. Thus, while connection and isolation were examined separately, participants' experiences suggest that they are not simply opposites, but rather, they are fluid, interacting and context‐dependent. Current outcome measures may fail to capture the complexities identified in this review, including the interacting and fluctuating relational and contextual dimensions that shape experiences of connection and isolation. Future research should build on these findings by addressing the aforementioned limitations and examining how these constructs interact and evolve post‐ABI. A deeper understanding of these dynamics could support the development or adaptation of more valid, experience‐aligned outcome measures.

Moreover, while our data identified key outcomes, contextual influences and highlighted interconnections between themes, the data did not support an analysis of how these elements interact or evolve as part of a broader process. To build on this study, future research should empirically investigate how factors such as stigma, identity and service or support networks align in ways that promote or inhibit social connection and isolation. It is also important to examine why some individuals avoid social connections while others are more receptive by investigating the interplay between individual, contextual and environmental influences. Qualitative studies could clarify how these mechanisms operate over time, for example, through in‐depth interviews or participatory methods with different groups of people with ABI in diverse contexts. Approaches such as a realist evaluation could help uncover the context–mechanism–outcome configurations [92] that shape these dynamic experiences and provide insight for the development or adaptation of more sensitive outcome measures and interventions.

While these considerations are important for advancing research, the findings regarding the key challenges faced and valued outcomes can also aid intervention design. Key areas for intervention include communication training, facilitating peer interactions and engagement in meaningful activities. Given the widespread nature of isolation post‐ABI, rehabilitation must incorporate support for social needs into standard care to fully promote recovery [7]. However, given the findings regarding the prevalence of stigma and exclusion [40], the underlying mechanisms of isolation must also be addressed through societal advancements [93]. For example, stereotypes could be dismantled through increased ABI awareness [94], so ABI‐related impairments are not associated with diminished intelligence or humanity.

## 5 Conclusions

This review explored the complex and varied experiences of social connection and isolation among adults with ABI. While meaningful connections, characterised by understanding, acceptance, reciprocity, emotional support and shared experiences, can promote identity and value reconstruction, such opportunities are not equally accessible. Environmental factors, such as stigma and limited service access, interact with ABI‐related impairments and reinforce isolation. These findings underscore the need for future research on appropriate outcome measures and interventions that address individual and structural influences, while also examining how connection and isolation evolve and interact across contexts.

## Author Contributions

**Jordan Ayden:** conceptualisation, methodology, formal analysis, validation, data curation, writing – original draft, writing – review and editing. **María‐José Bracho‐Ponce:** methodology. **Julia Ajayi:** validation, supervision, conceptualisation, methodology. **Sarah Hanson:** supervision, methodology, validation, writing – review and editing, conceptualisation. **Fergus Gracey:** supervision, methodology, validation, writing – review and editing, conceptualisation.

## Conflicts of Interest

The authors declare no conflicts of interest.

## Supporting information

Supplementary_file_1_search_strategy.

Supplementary_file_2_JBI_quality_ratings.

## Data Availability

Data is available on reasonable request.
