# Supplementary material for: The Experiences of Social Connection and Isolation in Adults With Acquired Brain Injury: A Qualitative Systematic Review and Metasynthesis
Source: Health Expect. 2025 Sep 8;28(5):e70420. doi: 10.1111/hex.70420 (PMC12415711; doi:10.1111/hex.70420)
Supplement: Supplementary file 1 — Supplementary_file_1_search_strategy. [file HEX-28-e70420-s001.docx]

PsychInfo search strategy

| S1 | TI ("brain injur*") OR AB ("brain injur*") |
| --- | --- |
| S2 | TI (ABI) OR AB (ABI) |
| S3 | TI (TBI) OR AB (TBI) |
| S4 | TI (stroke) OR AB (stroke) |
| S5 | TI ("brain h#emorrhage") OR AB ("brain h#emorrhage") |
| S6 | TI ("brain tumo#r") OR AB ("brain tumo#r") |
| S7 | TI ("brain infect*") OR AB ("brain infect*") |
| S8 | TI (encephalitis) OR AB (encephalitis) |
| S9 | TI ("head injur*") OR AB ("head injur*") |
| S10 | TI ("head trauma") OR AB ("head trauma") |
| S11 | TI ("cerebrovascular accident") OR AB ("cerebrovascular accident") |
| S12 | TI (CVA) OR AB (CVA) |
| S13 | TI ("brain aneurysm") OR AB ("brain aneurysm") |
| S14 | TI ("carbon monoxide poisoning") OR AB ("carbon monoxide poisoning") |
| S15 | TI (hydrocephalus) OR AB (hydrocephalus) |
| S16 | TI (hypoxi*) OR AB (hypoxi*) |
| S17 | TI (anoxi*) OR AB (anoxi*) |
| S18 | TI (meningitis) OR AB (meningitis) |
| S19 | DE "Brain Injur*" OR DE "Traumatic Brain Injury" |
| S20 | DE "Brain Damage" OR DE "Periventricular Leukomalacia" |
| S21 | DE "Cerebrovascular Accidents" |
| S22 | DE "Brain Lesions (Disorders)" |
| S23 | S1 OR S2 OR S3 OR S4 OR S5 OR S6 OR S7 OR S8 OR S9 OR S10 OR S11 OR S12 OR S13 OR S14 OR S15 OR S16 OR S17 OR S18 OR S19 OR S20 OR S21 OR S22 |
| S24 | TI ("social network*") OR AB ("social network*") |
| S25 | TI ("social isolat*") OR AB ("social isolat*") |
| S26 | TI (lonel*) OR AB (lonel*) |
| S27 | TI ("social connect*") OR AB ("social connect*") |
| S28 | TI (friend*) OR AB (friend*) |
| S29 | TI ("social inclus*") OR AB ("social inclus*") |
| S30 | TI ("social participat*") OR AB ("social participat*") |
| S31 | TI (befriending) OR AB (befriending) |
| S32 | TI ("social support*") OR AB ("social support*") |
| S33 | TI ("community integration") OR AB ("community integration") |
| S34 | TI ("social activit*") OR AB ("social activit*") |
| S35 | TI ("community group*") OR AB ("community group*") |
| S36 | AB ("Social Inclusion*") OR TI ("Social Inclusion*") |
| S37 | AB (belonging*) OR TI (belonging*) |
| S38 | DE "Social Isolation" |
| S39 | DE "Social Connectedness" |
| S40 | DE "Belonging" |
| S41 | DE "Loneliness" |
| S42 | DE "Social Networks" OR DE "Online Social Networks" |
| S43 | DE "Social Inclusion" |
| S44 | DE "Participation" |
| S45 | DE "Friendship" |
| S46 | DE "Social Support" |
| S47 | S24 OR S25 OR S26 S27 OR S28 OR S29 OR S30 OR S31 OR S31 OR S32 OR S33 OR S34 OR S35 OR S36 OR S37 OR S38 OR S39 OR S40 OR S41 OR S42 OR S43 OR S44 OR S45 OR S46 |
| S48 | AB qualitative OR TI qualitative |
| S49 | AB (”case stud*” OR case-stud*) OR TI (”case stud*” OR case-stud*) |
| S50 | AB “grounded theory” OR TI “grounded theory” |
| S51 | AB (phenomenolog* or “lived experience”) OR TI (phenomenolog* or “lived experience”) |
| S52 | AB “discourse analysis” OR TI “discourse analysis” |
| S53 | AB ethnograph* OR TI ethnograph* |
| S54 | AB narrative OR TI narrative |
| S55 | AB “content analysis” OR TI “content analysis” |
| S56 | AB thematic OR TI thematic |
| S57 | AB “focus group*” OR TI “focus group*” |
| S58 | AB “constant comparison” OR TI “constant comparison” |
| S59 | AB interpretive OR TI interpretive |
| S60 | AB hermeneutic* OR TI hermeneutic* |
| S61 | (DE “Qualitative Research”) OR (DE “Hermeneutic*”) OR (DE “Grounded Theory”) |
| S62 | S48 OR S49 OR S50 OR S51 OR S52 OR S53 OR S54 OR S55 OR S56 OR S57 OR S58 OR S59 OR S60 OR S61 |
| S63 | S23 AND S47 AND S62 |
