# Supplementary material for: The Experiences of Social Connection and Isolation in Adults With Acquired Brain Injury: A Qualitative Systematic Review and Metasynthesis
Source: Health Expect. 2025 Sep 8;28(5):e70420. doi: 10.1111/hex.70420 (PMC12415711; doi:10.1111/hex.70420)
Supplement: Supplementary file 2 — Supplementary_file_2_JBI_quality_ratings. [file HEX-28-e70420-s002.docx]

JBI quality ratings

| Reference | JBI Critical Appraisal Checklist for Qualitative Research question number | | | | | | | | | | Total score | Quality of contribution to the review |
| --- | --- | --- | --- | --- | --- | --- | --- | --- | --- | --- | --- | --- |
|  | 1 | 2 | 3 | 4 | 5 | 6 | 7 | 8 | 9 | 10 |  |  |
| Allen et al., 2022 | Y | Y | Y | Y | Y | N | Y | Y | Y | Y | 9 | Central |
| Archer et al., 2023 | U | Y | Y | Y | Y | N | Y | Y | N | Y | 7 | Core |
| Brady et al., 2011 | U | U | Y | U | N | N | N | Y | Y | N | 3 | Central |
| Brown et al., 2013 | Y | Y | Y | Y | Y | N | N | Y | Y | Y | 8 | Core |
| Carulli et al., 2018 | Y | Y | Y | Y | Y | N | Y | Y | U | N | 7 | Central |
| Dickson et al., 2008 | N | U | Y | N | N | N | N | Y | Y | Y | 4 | Peripheral |
| Douglas, 2020 | Y | Y | Y | Y | Y | U | N | Y | Y | Y | 8 | Core |
| Douglas, 2013 | Y | Y | Y | Y | Y | N | Y | Y | Y | Y | 9 | Central |
| Ford et al., 2024 | U | Y | Y | Y | Y | N | Y | Y | Y | Y | 8 | Core |
| Haun et al., 2007 | U | Y | Y | Y | Y | N | N | Y | U | Y | 6 | Core |
| Jumisko et al., 2008 | Y | Y | Y | Y | Y | N | Y | Y | Y | Y | 9 | Central |
| Kersey et al., 2024 | U | Y | Y | Y | Y | Y | Y | Y | Y | Y | 9 | Central |
| Kubina et al., 2013 | Y | Y | Y | Y | Y | N | Y | Y | Y | Y | 9 | Peripheral |
| Leahy et al., 2016 | Y | Y | Y | Y | Y | N | N | Y | Y | Y | 8 | Peripheral |
| Lefebvre et al., 2008 | U | Y | Y | Y | N | N | N | Y | Y | Y | 6 | Peripheral |
| Lowe et al., 2021 | U | Y | Y | Y | Y | Y | Y | Y | Y | Y | 9 | Central |
| Lynch et al., 2008 | U | Y | Y | Y | Y | N | N | Y | Y | Y | 7 | Peripheral |
| Manning et al., 2021 | Y | Y | Y | Y | Y | Y | N | Y | Y | Y | 9 | Central |
| Martin et al., 2015 | Y | Y | Y | Y | Y | N | Y | Y | Y | Y | 9 | Peripheral |
| Matérne et al., 2022 | U | Y | Y | Y | Y | Y | Y | Y | Y | Y | 9 | Central |
| May et al., 2023 | Y | U | Y | Y | Y | Y | Y | Y | Y | Y | 9 | Central |
| Moss et al., 2022 | U | Y | Y | Y | Y | N | Y | Y | Y | Y | 8 | Peripheral |
| Muldoon et al., 2018 | U | Y | Y | Y | Y | N | N | Y | Y | Y | 7 | Central |
| Murray & Harrison, 2004 | Y | Y | Y | Y | Y | N | N | Y | U | Y | 7 | Central |
| Nätterlund, 2010 | U | Y | Y | Y | Y | N | N | Y | Y | Y | 7 | Peripheral |
| Nichols & Kosciulek, 2014 | Y | U | U | N | N | U | U | U | Y | Y | 3 | Central |
| Northcott & Hilari, 2011 | U | Y | Y | Y | Y | N | Y | Y | Y | Y | 8 | Central |
| Northcott & Hilari, 2018 | U | Y | Y | Y | Y | Y | N | Y | Y | Y | 8 | Central |
| Pallesen et al., 2019 | Y | Y | Y | Y | Y | N | N | Y | Y | Y | 8 | Peripheral |
| Salas et al., 2018 | N | Y | Y | Y | Y | N | U | Y | N | Y | 6 | Core |
| Sathananthan et al., 2024 | Y | Y | Y | Y | Y | N | N | Y | Y | Y | 8 | Peripheral |
| Theeke et al., 2017 | Y | Y | Y | Y | Y | N | N | Y | Y | Y | 8 | Peripheral |
| Törnbom et al., 2019a | U | Y | Y | Y | Y | Y | N | Y | Y | Y | 8 | Peripheral |
| Törnbom et al., 2019b | U | Y | Y | Y | Y | Y | N | Y | Y | Y | 8 | Peripheral |
| Turner et al., 2009 | Y | Y | Y | Y | Y | N | Y | Y | Y | Y | 9 | Peripheral |
| von Vogelsang et al., 2024 | U | Y | Y | Y | Y | N | Y | Y | Y | Y | 8 | Peripheral |
| Yang et al., 2022 | U | Y | Y | Y | Y | N | N | Y | Y | Y | 7 | Peripheral |
